# Core muscle endurance and psychosocial factors affecting functional mobility in chronic low back pain: cross-sectional results

**DOI:** 10.7717/peerj.20372

**Published:** 2025-12-19

**Authors:** Ravi Shankar Reddy, Mastour Saeed Alshahrani, Hani Hassan Alnakhli, Batool Abdulelah Alkhamis, Ghada Mohamed Koura, Debjani Mukherjee, Raee S. Alqhtani, Faisal M. Alyazedi, Amal Fahad Al Mukhid, Saeed Ahmed M. Alhudiry

**Affiliations:** 1 Physical Therapy Program, Medical Rehabilitation Sciences, King Khalid University, Abha, Aseer, Saudi Arabia; 2 Department of Physical Therapy, Najran University, Najran, Saudi Arabia; 3 Department of Physical Therapy, Prince Sultan Military College of Health Sciences, Dhahran, Saudi Arabia; 4 Department of Physical Therapy, Abha International Private Hospital, Abha, Aseer, Saudi Arabia

**Keywords:** Chronic low back pain, Core endurance, Functional mobility, Psychosocial factors, Pain catastrophizing, Fear-avoidance beliefs

## Abstract

**Background:**

The functional limitations associated with chronic low back pain (CLBP) are substantial and are highly correlated with core muscle endurance and psychosocial factors. Core muscle endurance is known to affect physical performance, whereas pain catastrophizing and fear avoidance beliefs can contribute to disability. A comprehensive understanding of the interrelated impact of these factors is critical for developing effective rehabilitative strategies. The study aimed to (1) examine the correlation between core muscle endurance and functional mobility in CLBP subjects and (2) explore the influence of psychosocial factors, pain catastrophizing, and fear-avoidance beliefs on that correlation.

**Methods:**

A cross-sectional study was conducted on 136 adults; core muscle endurance was measured by plank, side-bridge, and back extensor tests. The timed up and go (TUG) and five times sit-to-stand test (5TSTS) were utilized to evaluate functional mobility. Pain intensity was assessed using the visual analog scale (VAS), and disability using the Oswestry disability index (ODI). Psychosocial factors were measured using the pain catastrophizing scale (PCS) and the fear-avoidance beliefs questionnaire (FABQ).

**Results:**

Core muscle endurance measures were moderately correlated with functional mobility outcomes (TUG: *r* = −0.42 to −0.50, *p* < 0.05; 5TSTS: *r* =  − 0.35 to −0.43, *p* < 0.05). Hierarchical regression showed muscle endurance accounted for 32% of the variance in mobility, while adding psychosocial factors increased the adjusted R^2^ to 0.52 (*p* < 0.05). High-psychosocial-risk subgroups analyzed within the study had poorer mobility (TUG: 9.45 ± 1.32 *vs.* 7.89 ± 1.21 s; *p* = 0.001).

**Conclusion:**

Psychosocial factors and endurance of the core muscles in CLBP can significantly predict functional mobility. Integrating physical and psychological rehabilitation strategies might improve mobility outcomes, bolster human dignity, and enhance quality of life.

## Introduction

Chronic low back pain (CLBP) is a highly prevalent syndrome that has a significant economic impact on individuals and health systems worldwide (Fatoye et al., 2023). CLBP imposes a substantial economic burden on both affected individuals, through lost productivity and treatment costs, and healthcare systems due to prolonged care needs (Kahere et al., 2022). Spinal stabilization and optimal movement patterns during functional activities depend on the core musculature, which includes the trunk and pelvic muscle groups (Zemková & Zapletalová, 2022). Core muscle endurance is crucial in determining functional mobility in individuals with CLBP. This is supported by prior literature, which highlights that insufficient endurance of core muscles contributes to impaired postural control and biomechanical instability in individuals with CLBP (Alshahrani, Reddy & Ravi, 2025; Fallahasady et al., 2022). This measure reflects the muscles’ capacity to endure a sustained stimulus (Li et al., 2025). It has been suggested that impairments in core muscle endurance may lead to biomechanical instability and compensatory movement strategies, which enhance pain sensitivity and further restrict functional capacity (Li et al., 2025).

Multiple studies have established a significant correlation between core muscular endurance and functional mobility in individuals with CLBP (Özyurt, Aksoy & Özkaya, 2024). Core muscle endurance assessments, such as the plank, side-bridge, and back extensor endurance tests, have historically been employed to evaluate trunk stability and its impact on lower extremity performance in functional activities (Behm, Daneshjoo & Alizadeh, 2022). These tests evaluate certain muscle groups’ strength and endurance and indirectly assess the lumbar-pelvic complex’s overall stability (Gies, 2021). Functional mobility assessments such as the timed up and go test (TUG) and the five times sit-to-stand test (5TSTS) are commonly employed to evaluate dynamic mobility and transitions related to activities of daily living (Albalwi & Alharbi, 2023). Numerous studies have indicated that persons with greater core muscle endurance have superior performance in functional mobility assessments (Saeterbakken et al., 2022). Nonetheless, the precise processes underlying this association and its therapeutic importance remain inadequately defined, especially within CLBP populations. This limitation is particularly noted in the domain of CLBP rehabilitation and functional performance.

In addition to physical issues, psychosocial elements significantly influence functional outcomes in individuals with CLBP (Ho et al., 2022). Pain catastrophizing and fear-avoidant attitudes are among the most extensively researched psychosocial phenomena in this domain (Cocciuti, 2021). These constructs were selected based on their robust empirical associations with disability, functional limitation, and treatment outcomes in individuals with chronic pain. Pain catastrophizing reflects a maladaptive cognitive-emotional response that amplifies the perception of pain and predicts adverse physical and psychological outcomes. Similarly, fear-avoidant beliefs contribute to physical deconditioning and reduced activity levels, both of which are relevant to the mobility deficits observed in CLBP. These variables are also key components of the fear-avoidance model, a widely accepted framework in pain rehabilitation research. Pain catastrophizing is characterized by an overly negative cognitive framework regarding pain events (Petrini & Arendt-Nielsen, 2020). Fear-avoidance beliefs pertain to the inclination to evade physical action due to the apprehension of pain or reinjury (Jaén Parrilla, 2022). Both have been linked to greater disability and poorer functional results in chronic pain sufferers. These psychosocial factors independently affect functional impairment and may also interact with physical characteristics, such as core muscle endurance, influencing mobility (Zhao et al., 2021). An individual exhibiting heightened catastrophizing or fear-avoidance attitudes will establish a maladaptive movement pattern, while a person with restricted physical activity will encounter diminished core stability and endurance effects (Khan, 2020).

Despite the growing recognition of the mutual connection between physical and psychosocial factors in CLBP, research investigating their combined impact on functional mobility remains limited (Nordstoga, 2020). Most prior research has concentrated on either the physical or psychological dimensions. Consequently, a significant gap persists in comprehending the influence of core muscle endurance and psychological factors on mobility outcomes (Johnston, 2021). Furthermore, while the associations among core muscle endurance, psychosocial factors, and functional mobility have been examined separately, the moderating influence of psychosocial factors in this relationship remains unverified (Johnston, 2021). Addressing this gap is essential for developing more effective and comprehensive rehabilitation procedures that include both the physical and psychological dimensions of CLBP.

This study aims to investigate the correlation between core muscle endurance and functional mobility in patients with CLBP while also examining the influence of psychosocial factors, specifically pain catastrophizing and fear-avoidance attitudes on this association. The study hypothesized that (1) greater core muscle endurance would be associated with improved functional mobility outcomes, as measured by TUG and 5TSTS; (2) higher levels of pain catastrophizing and fear-avoidance beliefs would be independently associated with poorer mobility performance; and (3) psychosocial factors would moderate the relationship between core muscle endurance and mobility, such that individuals with higher psychosocial distress would exhibit weaker associations between physical endurance and functional outcomes.

## Methods

### Ethics, setting, and design

This cross-sectional study was performed from April 2023 to March 2024 at the Department of Rehabilitation Medicine in a tertiary care hospital specializing in musculoskeletal disorders in Abha, Saudi Arabia. The Research Ethics Committee of KKU, DSR (REC# 234-2024) gave ethical clearance before the study. All individuals provided their written informed consent before participation. All study procedures were conducted in accordance with the ethical principles outlined in the Declaration of Helsinki.

### Participants

Participants were selected using purposive sampling to ensure the sample represented the study’s target population. This sampling method was employed to ensure that all participants met the diagnostic criteria for CLBP and were functionally able to perform the endurance and mobility tasks, which would not have been feasible under a random sampling approach due to expected heterogeneity in eligibility. Participants were recruited *via* direct provider referrals and informational posters in the outpatient department. The diagnosis of CLBP was made by clinical criteria (pain localized to the region between the lower rib margin and gluteal folds for more than three months without a specific identifiable pathology, such as fracture, infection, or malignancy) (Mohd Isa et al., 2022). Participants were enrolled upon referral from a consulting physician or physiotherapist and were screened using inclusion and exclusion criteria by the research team. Body mass index (BMI) was calculated using weight and height measurements obtained *via* a calibrated digital scale and stadiometer. Duration of pain was assessed through participant self-report. Pain intensity was measured using a 10-point Visual Analogue Scale (VAS), and disability was assessed using the Oswestry Disability Index (ODI). Inclusion criteria were age 18 to 65 years, clinically diagnosed CLBP, BMI of 18 to 35 kg/m^2^, and ability to perform basic functional mobility tasks, including walking and transitioning from sitting to standing. Participants were eligible if they were medically stable and could provide written informed consent. Basic functional abilities such as walking and sit-to-stand were confirmed during the screening process by direct observation from a licensed physiotherapist. Medically stable was defined as free from acute illness, hemodynamically stable, and not undergoing any ongoing treatment that would contraindicate physical assessments. Exclusion criteria were as follows: neurological disorders (radiculopathy, myelopathy), spine or abdominal surgery in the previous 6 months, significant musculoskeletal injury in the lower limbs, or any other comorbidity that could hinder functional mobility (cardiovascular disease or advanced osteoarthritis). Those who were cognitively impaired or had active psychiatric conditions that could impede their capacity for completing the assessments were also excluded from the study.

Participant use of pain medications was recorded during intake and considered in subgroup sensitivity analyses to account for its potential influence on pain perception and mobility performance.

### Anthropometric and clinical outcomes

Anthropometric characteristics included BMI (kg/m^2^), calculated from self-reported height and weight. Anthropometric characteristics included BMI (kg/m^2^), calculated from height and weight measurements obtained *via* a calibrated digital scale and stadiometer. Clinical outcomes assessed were pain intensity using the VAS (0–10 cm), disability using the ODI (%), and pain duration in months. These measures were collected during the initial assessment phase to characterize the sample and explore associations with functional mobility outcomes.

### Sample size estimation

The sample size was calculated using G*Power software (version 3.1.9.7) based on a moderate effect size (*r* = 0.3) derived from prior research (Fallahasady et al., 2022), who examined the relationship between core muscle endurance and functional performance in a musculoskeletal population with similar demographics and methodological design, with a significance level of 0.05 and a power of 0.80. A 10% dropout rate was accounted for, resulting in a target sample size of 136 participants.

### Core muscle endurance

Core muscle endurance was assessed using three validated static endurance tests: the plank endurance test (Choi, Kim & Cynn, 2021), the side-bridge endurance test (Hajmirzaei Tafreshi, Minoonejad & Seidi, 2023), and the back extensor endurance test (Clael et al., 2021). All measurements were taken under optimal clinical conditions in a controlled laboratory setting. Trained physiotherapists supervised all assessments, providing detailed verbal instructions to participants and monitoring their movements to ensure proper execution. Participants performed a brief warm-up prior to testing, including light stretching and low-intensity movements for muscle preparation. The warm-up lasted about five minutes and involved dynamic trunk stretches, arm swings, heel raises, and low-intensity walking. The physiotherapist demonstrated each test and allowed participants a moment to briefly confirm their understanding of the protocol to ensure proper performance.

**Figure 1:**
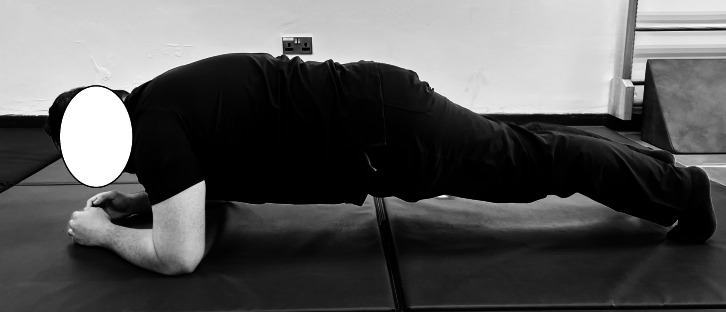
Standard plank endurance test position. Participant maintains a straight, aligned posture with forearms under shoulders to assess anterior core endurance through isometric hold.

In the plank endurance test (Choi, Kim & Cynn, 2021), participants were instructed to lie prone on a mat with their elbows directly beneath their shoulders and their forearms flat on the ground (Fig. 1). The feet were positioned at hip-width, with the toes flexed to support the body. Participants were then directed to lift their bodies off the mat, maintaining a straight line from head to heels while engaging their core muscles. The physiotherapist emphasized the importance of keeping the hips level and avoiding sagging or arching the lower back. Timing started when the participant assumed the correct plank position and ended when the position was no longer held or the participant voluntarily ended the test. Timing was recorded using a digital stopwatch accurate to 0.01 s.

**Figure 2:**
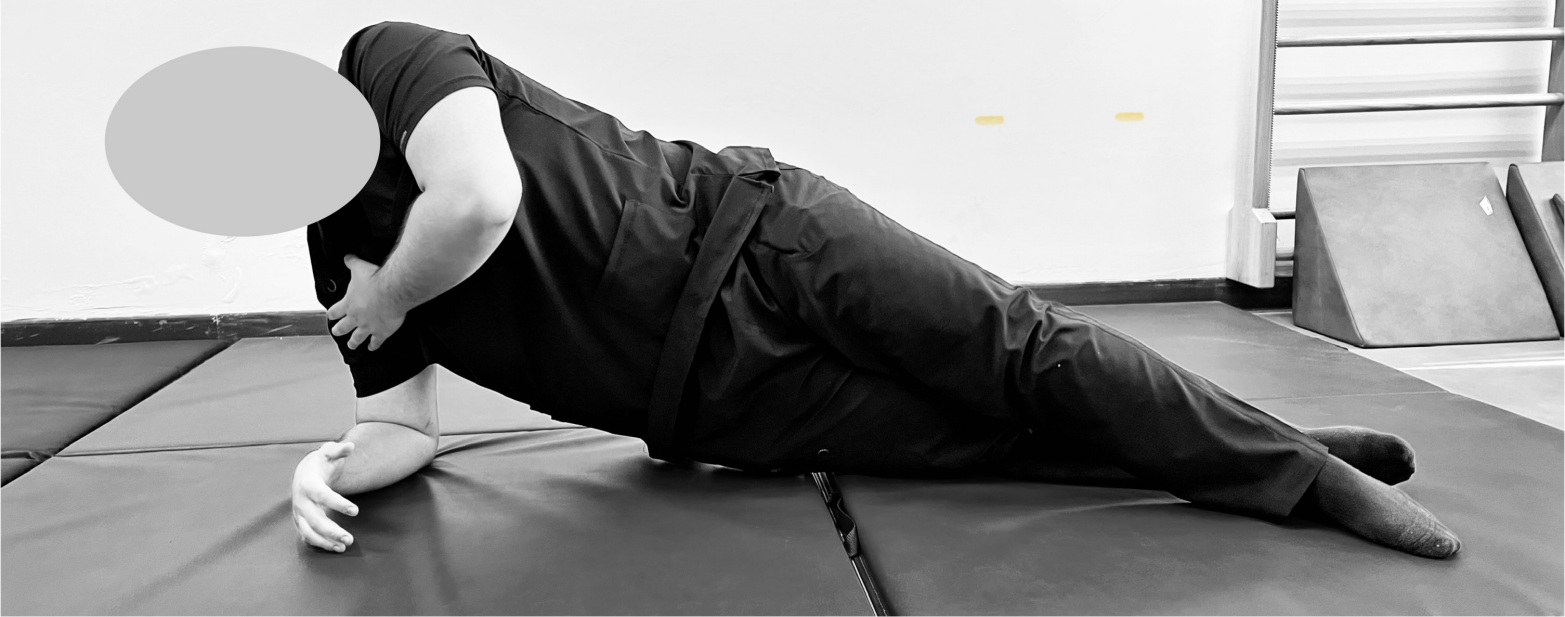
Standard side-bridge endurance test position. Participant demonstrates the side-bridge test, maintaining lateral alignment with the elbow beneath the shoulder to assess oblique and lateral core endurance through isometric hold.

The side-bridge endurance test (Hajmirzaei Tafreshi, Minoonejad & Seidi, 2023) assessed lateral core muscle endurance. Participants were instructed to assume a side-lying position, with legs extended and aligned, the elbow of the lower arm positioned directly beneath the shoulder, and the forearm perpendicular to the body (Fig. 2). The opposite arm rested alongside the torso. The physiotherapist then directed participants to lift their hips off the mat, create a straight line from head to feet, and engage their oblique muscles while preventing any rotation or twisting of the torso. Timing started when the correct side-bridge position was achieved and ended when the position could no longer be maintained or the participant voluntarily stopped the test. The average of the two times from both sides was calculated for analysis.

**Figure 3:**
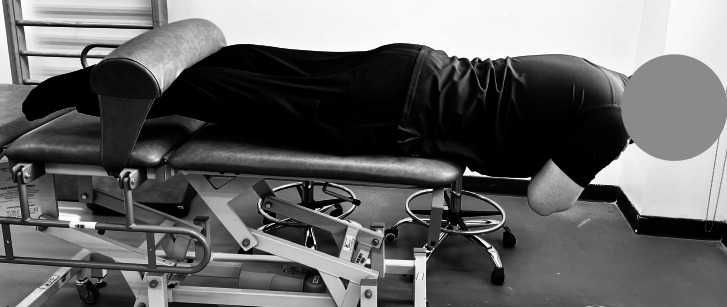
Standard back extensor endurance test position. Participant maintains a horizontal trunk position with the lower body secured to assess isometric endurance of the back extensors, commonly used in evaluating posterior core strength.

The back extensor endurance test was employed to evaluate the endurance of the back extensor muscles (Clael et al., 2021). The participants were prone on a plinth, with their lower bodies secured to an adjustable padded bench (Fig. 3). The iliac crest was aligned with the edge of the support platform, and the lower limbs were bound with adjustable straps or restrained by an attendant. The thumbs-up gesture was designated for participants instructed to cross their arms over their chests. Subsequently, the physiotherapist demonstrated the proper alignment for elevating the trunk from the mat, ensuring the upper body remained parallel to the plinth and perpendicular to the lower body. Participants were given a brief time to assume the correct position, and support was provided as needed to minimize premature fatigue before timing began. Participants were additionally directed to engage their back-extensor muscles while preventing cervical flexion. The timing commenced upon achieving the horizontal posture and concluded when the individual could no longer sustain the position or voluntarily ended the test. Timing was recorded using the same digital stopwatch method as the other endurance tests. The duration was quantified in seconds. Regardless of the tests conducted, standard verbal reinforcement was provided to maintain the position for as long as feasible. Safety protocols were used, including halting participants upon reporting pain or exhibiting indicators of discomfort or perilous postural deviations. Additionally, for each test, aggregated outputs were compiled on a standardized datasheet, ensuring uniformity in the output format utilized by all participants.

### Functional mobility

Two widely recognized and standardized assessments were employed to evaluate functional mobility: the TUG and the 5TSTS (Trombini-Souza et al., 2023). The TUG necessitated that participants transition from a seated to a standing position, traverse a distance of three meters, execute a turn, and return to a seated position; the total duration to complete the task was recorded in seconds (Trombini-Souza et al., 2023). The 5TSTS test required participants to rise from a seated posture and thereafter return to sitting for five repeats, with the total duration measured in seconds. The assessments were performed on level, non-slippery surfaces, and participants were provided with verbal instructions and demonstrations before the testing. A standard armless chair, 43 cm in height, was used, positioned against a wall to prevent displacement during the test. To minimize variability, the measures were conducted twice, and the mean score was utilized for analysis. Timing was recorded using a digital stopwatch. To ensure consistency across assessors, two trained physiotherapists independently scored the timing-based tests (TUG and 5TSTS). Inter-rater reliability was evaluated on a random 15% subsample, with intra-class correlation coefficients exceeding 0.90 for all mobility measures, indicating excellent agreement.

### Psychological factors

Psychosocial variables were assessed *via* validated self-report measures. Pain catastrophizing was assessed *via* the pain catastrophizing scale (PCS), which assesses cognitive and emotional aspects of catastrophizing/risk factors, including rumination, magnification, and helplessness, with higher scores indicative of greater catastrophizing (Franchignoni et al., 2022). The PCS was originally developed and validated by Sullivan, Bishop & Pivik (1995), demonstrating strong internal consistency and predictive validity in chronic pain populations. For subgroup analyses, high-risk participants were defined as those with PCS scores ≥ 24, a threshold supported by prior validation studies as indicating clinically significant catastrophizing (Sullivan, Bishop & Pivik, 1995). The fear-avoidance beliefs questionnaire (FABQ) was employed to assess fear-avoidance beliefs related to physical activity and work activity, with higher scores representing greater avoidance tendencies (Franchignoni et al., 2022). The FABQ was originally introduced by Waddell et al. (1993) as a measure of patients’ beliefs about how physical activity and work may influence their pain, with established reliability and construct validity. High-risk categorization for FABQ followed previously reported thresholds, with scores ≥ 20 indicating elevated fear-avoidant beliefs of clinical importance (Waddell et al., 1993). The PCS and FABQ were administered in English using their validated full-length versions. Participants who required clarification received standardized explanations in their native language, provided by bilingual research staff trained in questionnaire administration. The questionnaires were administered in a standardized, distraction-free environment, with a trained physiotherapist present to clarify instructions when necessary. Participants completed the forms independently to minimize interviewer bias.

### Data analysis

Statistical analysis was conducted utilizing SPSS (version 24) and R software (version 4.2). We employed the Shapiro–Wilk test to assess the normality of the data distribution and verified its normality. Consequently, parametric statistical methods may be utilized. Regression assumptions were systematically verified. Linearity and homoscedasticity were assessed through inspection of standardized residual plots, and normality of residuals was confirmed *via* the Shapiro–Wilk test. Independence of errors was evaluated using the Durbin–Watson statistic. Multicollinearity was assessed with variance inflation factors (VIF), all of which were <2, indicating negligible collinearity even after inclusion of interaction terms. To strengthen model validity, hierarchical regression analyses additionally adjusted for age, sex, BMI, and pain duration as covariates. Participant demographics, core muscle endurance metrics, functional mobility results, and psychosocial factors were reported by descriptive statistics, encompassing means and standard deviations. Categorical variables, including sex, were summarized using frequency counts and percentages. Pearson’s correlation coefficient was employed to analyze the association between core muscular endurance (plank, side-bridge, and back extensor endurance) and functional mobility metrics (TUG test and 5TSST). The independent effects of core muscle endurance and psychosocial factors, assessed by the PCS and FABQ, on functional mobility were analyzed by hierarchical multiple regressions. Given that six correlation tests were performed (three endurance measures × two mobility outcomes), a Bonferroni adjustment was applied to reduce the risk of Type I error. The adjusted significance threshold was set at *p* < 0.008. In the hierarchical regression analysis, core muscle endurance measures were entered in the first step, followed by psychosocial factors (PCS and FABQ) in the second step, and interaction terms (core muscle endurance × PCS and core muscle endurance × FABQ) in the final step. This approach allowed us to assess the independent and interactive effects of core muscle endurance and psychosocial factors on functional mobility. In the final step, interaction terms were incorporated to investigate the moderating impacts of psychological variables. An independent samples *t*-test was conducted to assess functional mobility outcomes across high and low-psychosocial risk groups, followed by a one-way ANOVA to verify interaction effects.

**Table 1:** Demographic and clinical characteristics of participants (*n* = 136).

**Variables**	**Mean ± SD/Frequency (%)**
Age (years)	45.32 ± 10.54
Sex (Male/Female)	65 (47.79%)/71 (52.21%)
BMI (kg/m^2^)	26.78 ± 3.45
Duration of pain (months)	24.31 ± 8.67
Pain intensity (VAS, 0–10 cm)	5.45 ± 2.34
Disability (ODI, 0–100%)	35.21 ± 12.56
Pain catastrophizing scale (PCS)	21.45 ± 6.32
FABQ	23.12 ± 7.48
Plank endurance (seconds)	47.89 ± 15.67
Side-bridge endurance (seconds)	35.23 ± 12.45
Back extensor endurance (seconds)	70.15 ± 18.34
Timed up and go test (TUG, seconds)	8.32 ± 1.67
Five times sit-to-stand test (5TSTS, seconds)	11.45 ± 2.12

**Notes.**

Sex refers to biological classification (male/female).

Abbreviations BMI body mass index (kg/m^2^) VAS visual analog scale (0–10 cm) ODI Oswestry disability indexs (%) PCS pain catastrophizing scale (score) FABQ fear-avoidance beliefs questionnaire (score) TUG timed up and go test (seconds) 5TSTS five times sit-to-stand test (seconds)

## Results

The demographic, anthropometric, and clinical characteristics of the participants (Table 1) indicated that the cohort is middle-aged (mean age 45.32 ± 10.54 years), with a nearly equal distribution of males and females. The average BMI was classified as overweight (26.78 ± 3.45 kg/m^2^), and the average duration of pain was 24.31 ± 8.67 months. The pain intensity and disability scores indicated moderate chronic pain impact, with a VAS score of 5.45 ± 2.34 and an ODI score of 35.21 ± 12.56%. Results indicated moderate levels of catastrophizing (21.45 ± 6.32) and fear-avoidant attitudes (23.12 ± 7.48) in individuals with CLBP. Core muscle endurance assessments exhibited significant diversity in performance: mean endurance durations were 47.89 ± 15.67 s” for the plank, 35.23 ± 12.45 s” for the side-bridge, and 70.15 ± 18.34 s” for the back-extensor endurance tests, respectively. Functional mobility assessments produced a mean TUG time of 8.32 ± 1.67 s”, whereas the mean 5TSTS time was 11.45 ± 2.12 s”.

**Figure 4:**
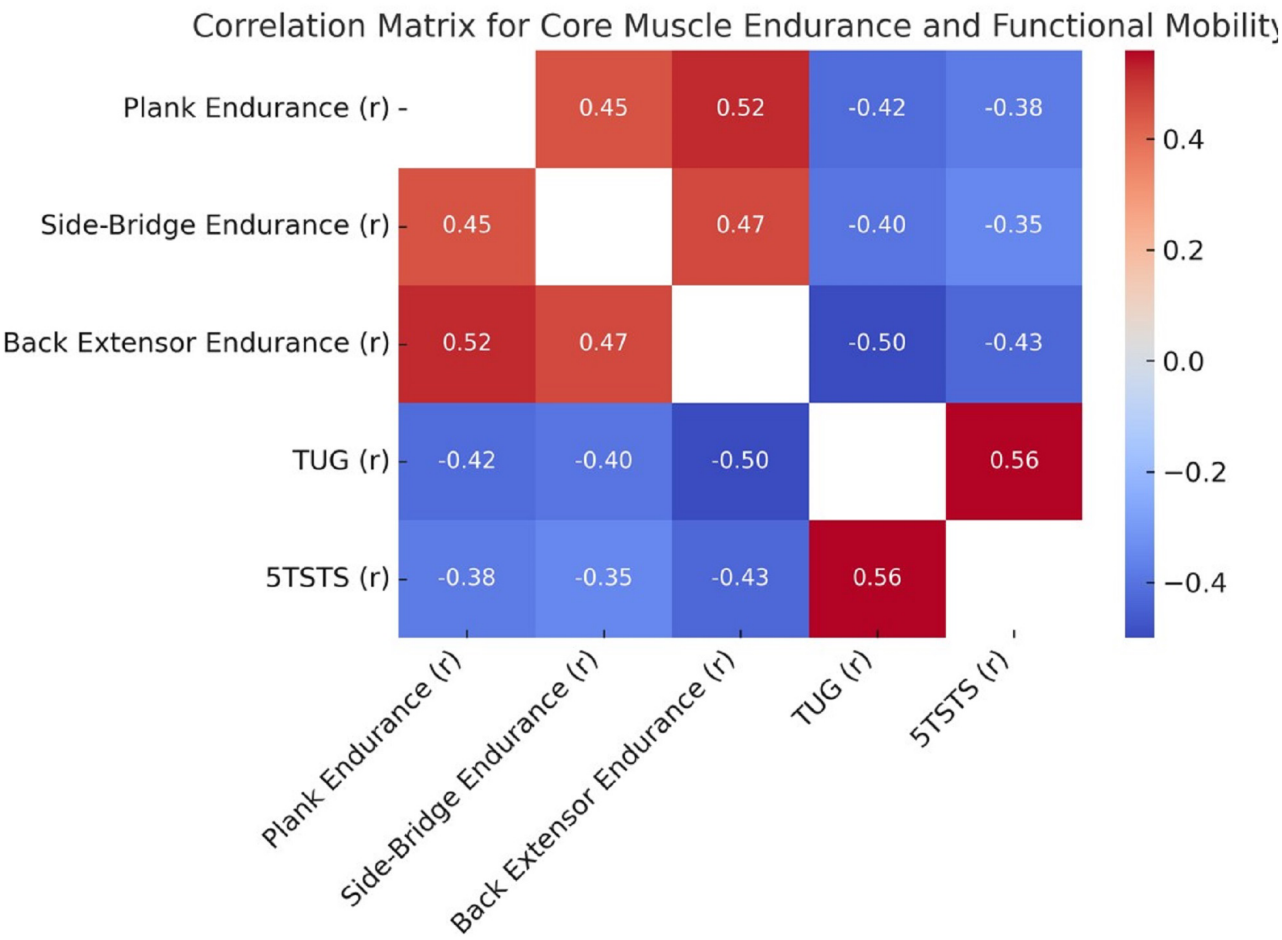
Correlation matrix for core muscle endurance and functional mobility. Heatmap illustrating Pearson’s correlation coefficients between core endurance tests and mobility outcomes. Stronger core endurance is associated with better functional mobility, as shown by inverse correlations with TUG and 5TSTS.

As shown in Fig. 4, core muscle endurance measures—back extensor endurance showed the strongest negative correlations with both the TUG test (*r* = −0.50) and the 5TSTS test (*r* = −0.43). Plank and side-bridge endurance were inversely correlated with TUG (*r* = −0.42 and −0.40) and 5TSTS (*r* = −0.38 and −0.35), respectively.

Table 2 shows that core muscle endurance significantly predicted functional mobility, explaining 32% of the variance in the first step of the hierarchical regression model. The inclusion of psychosocial factors (PCS and FABQ) increased the model’s adjusted R^2^ by 13% (PCS: *p* = 0.001; FABQ: *p* = 0.023). Interaction terms added a further 7% variance (PCS × core muscle endurance: *p* = 0.002; FABQ × core muscle endurance: *p* = 0.015).

The multivariable linear regression analysis identified core muscle endurance, psychosocial factors, and demographic variables as significant predictors of functional mobility outcomes (Table 3). Core muscle endurance was the strongest predictor, demonstrating a significant negative association with functional mobility times (β = −0.42, *p* = 0.001), contributing 15% to the adjusted R^2^. Among psychosocial factors, both the PCS (β = 0.35, *p* = 0.002) and the FABQ (β = 0.28, *p* = 0.007) were significant contributors, collectively explaining an additional 13% and 10% of the variance, respectively. Age (β = 0.12, *p* = 0.034) and BMI (β = 0.15, *p* = 0.021) were also significant predictors, although their contributions to the adjusted R^2^ were smaller. Biological sex (male/female) showed no significant association with functional mobility (β = −0.05, *p* = 0.186).

Psychosocial Risk Stratification: functional mobility in subgroup analysis varied according to psychosocial risk strata (Table 4). High-risk subgroups were defined as PCS ≥ 24 and FABQ ≥ 20, based on prior psychometric literature identifying these thresholds as indicative of clinically significant catastrophizing and fear-avoidant beliefs, respectively. Participants with higher PCS scored significantly slower TUG and 5TSTS (mean TUG (s”): 9.45 ± 1.32 *vs.* 11.11 ± 2.01, *p* = 0.001, mean 5TSTS (s”): 12.45 ± 2.12 *vs.* 13.88 ± 3.43, *p* = 0.002). Similarly, participants in the high FABQ group demonstrated slower TUG and 5TSTS times compared to the low FABQ group, with mean times of 9.78 ± 1.40 s and 12.87 ± 2.34 s, respectively (*p* = 0.001 for both tests).

**Table 2:** Hierarchical regression analysis showing the independent and interaction effects of core muscle endurance and psychosocial factors (PCS and FABQ) on functional mobility outcomes (TUG and 5TSTS).

**Model step**	**Predictor variables**	**Beta coefficient (β)**	**Standard error (SE)**	**Adjusted R^2^**	ΔR^2^	***p*-Value**
Step 1: Core Muscle Endurance	Core endurance	0.45	0.08	0.32	0.32	0.001
Step 2: + Psychosocial Factors (PCS, FABQ)	Core endurance, PCS, FABQ	0.38, −0.25, −0.18	0.09, 0.07, 0.06	0.45	0.13	0.001, 0.023, 0.035
Step 3: + Interaction Terms	Core endurance × PCS, Core endurance × FABQ	0.35, −0.20, −0.15	0.10, 0.08, 0.07	0.52	0.07	0.002, 0.015, 0.041

**Notes.**

PCS pain catastrophizing scale FABQ fear-avoidance beliefs questionnaire β Beta Coefficient SE standard error R^2^ Coefficient of Determination ΔR^2^ Change in R^2^ *p* *p*-value

**Table 3:** Multivariable linear regression for predicting functional mobility outcomes.

**Predictor variables**	**Beta coefficient (β)**	**Standard error (SE)**	**95% Confidence interval (CI)**	***t*-Value**	**Adjusted R^2^ contribution**	***p*-Value**
Core endurance (seconds)	−0.42	0.08	[−0.58, −0.26]	−5.25	0.15	0.001
PCS	0.35	0.09	[0.17, 0.53]	3.89	0.13	0.002
FABQ	0.28	0.07	[0.14, 0.42]	4.00	0.10	0.007
Age (years)	0.12	0.05	[0.02, 0.22]	2.40	0.05	0.034
BMI (kg/m^2^)	0.15	0.06	[0.03, 0.27]	2.50	0.06	0.021
Gender (Male = 0, Female = 1)	−0.05	0.04	[−0.13, 0.03]	−1.25	0.01	0.186

**Notes.**

PCS pain catastrophizing scale FABQ fear-avoidance beliefs questionnaire BMI body mass index β Beta Coefficient SE standard error CI confidence interval *t* *t*-Value Adjusted R^2^ Adjusted Coefficient of Determination

All beta coefficients are standardized. Positive coefficients for PCS and FABQ reflect their independent contributions to increased mobility times (poorer performance), consistent with theoretical expectations.

**Table 4:** Functional mobility outcomes (TUG and 5TSTS) among subgroups stratified by psychosocial risk. High-risk groups were defined as PCS ≥ 24 and FABQ ≥ 20, based on established clinical cut-off scores.

**Subgroups**	**N (sample size)**	**Mean TUG (seconds) ± SD**	**Mean 5TSTS (seconds) ± SD**	***t*-Value (TUG)**	***t*-Value (5TSTS)**	***p*-Value (TUG)**	***p*-Value (5TSTS)**
High PCS group	50	9.45 ± 1.32	12.45 ± 2.12	5.43	4.78	0.001	0.002
Low PCS group	86	7.89 ± 1.21	10.34 ± 1.85	–	–	–	–
High FABQ group	45	9.78 ± 1.40	12.87 ± 2.34	6.12	5.90	0.001	0.001
Low FABQ group	91	7.65 ± 1.18	10.12 ± 1.67	–	–	–	–

**Notes.**

PCS pain catastrophizing scale FABQ fear-avoidance beliefs questionnaire TUG timed up and go test 5TSTS five times sit-to-stand test N sample size SD standard deviation *t* *t*-Value *p* *p*-value

## Discussion

This study investigates the relationship between core muscle endurance and functional mobility among CLBP patients, as well as the moderating effect of psychosocial factors (pain catastrophizing and fear-avoidant beliefs). Significant relationships were identified between core muscle endurance measures and functional mobility outcomes, with higher values of core muscle endurance associated with better performance on TUG and 5TSTS. The PCS and FABQ contributed significantly individually in the hierarchical regression, which further demonstrates that psychosocial factors were independently associated with the outcomes of interest and moderated the relationship between core muscle endurance and mobility. Subgroup analysis additionally highlighted the influence of psychosocial risk, evidenced by a significantly slower TUG and 5TSTS time in the participants with higher PCS and FABQ scores relative to those with low psychosocial risk. These results further emphasize the relationship between physical and psychological factors on functional mobility and serve as a basis for focused interventions in this population.

The significant relationships observed between core muscle endurance and functional mobility outcomes in this study may be explained by the vital role of the core musculature in supporting trunk stiffness and lower extremity performance during dynamic movement (Junker & Stöggl, 2019; Nakagawa & Petersen, 2018). The moderate correlations of plank, side-bridge, and back extensor endurance tests suggest that these measures all evaluate potentially overlapping components of core stability and strength (Zemková, 2022). The most significant negative connection was observed between back extensor endurance and TUG timings, underscoring the importance of the posterior chain in mobilization activities that necessitate postural control and balance (Motimath, Neelgund & Chivate, 2019). Similarly, the slight negative correlations seen between all core muscle endurance metrics and 5TSTS timings indicate the role of core strength in transitions between seated and standing positions, necessitating trunk stabilization and effective force transmission through the lower extremities (Gonçalves de Oliveira et al., 2023). The findings underscore the biomechanical interrelation between core stability and lower extremity performance in a CLBP population, where compensatory processes may result in increased mobility limitations (Gonçalves de Oliveira et al., 2023). The results align with prior studies emphasizing the significant role of core muscle endurance in functional performance and mobility (Frizziero et al., 2021). The correlations with enhanced TUG and 5TSTS times may stem from an augmented capacity to sustain postural control during dynamic tasks attributable to greater core stability (Azharuddin & Zia, 2021). Steele, Bruce-Low & Smith (2014) identified the posterior chain, particularly back extensor endurance, as a principal predictor of mobility in individuals with lower back pain, corroborating the significant correlation shown in this study (Steele, Bruce-Low & Smith, 2014). Furthermore, research conducted by Wang et al. (2023) indicates that enhanced core muscle endurance reduces compensatory movements and energy inefficiencies during functional tasks, hence reinforcing the association between higher core muscle endurance and improved mobility outcomes (Wang et al., 2023). Collectively, these findings not only corroborate the current study’s results but also underscore the necessity of integrating core-specific training into rehabilitation programs to optimize enhancements in functional mobility and independence among individuals with CLBP.

Core muscular endurance and psychosocial factors are substantial determinants of functional mobility, as indicated by the hierarchical multiple regression analysis (Kehl et al., 2024). Core muscle endurance was recognized as a primary predictor, explaining 32% of the variance in mobility outcomes (Elabd, Oakley & Elabd, 2024). The incorporation of psychosocial variables, notably the PCS and FABQ, significantly enhanced the model’s explanatory power, resulting in an adjusted R^2^ of 0.52 with the inclusion of interaction terms. The findings demonstrate that psychosocial factors independently affect mobility and interact with physical traits, underscoring the interplay between physical and psychological domains as determinants of functional performance (Vianin, 2021). Multivariable linear regression indicated that a combination of core muscle endurance, psychosocial factors, and demographic variables accounts for a significant percentage of the variance in functional mobility (Akodu et al., 2024). The varying degrees of significant correlations between PCS and FABQ with functional outcomes highlight the essential impact of the psychosocial aspect (Alqhtani et al., 2023).

Previous research emphasizing the importance of physical and psychological factors generally treats the latter as complementary components in models of mobility impairment (Hernandez-Lucas et al., 2023). Fallahasady et al. (2022) identified core stability as an important factor affecting functional performance, correlating with the predictive capacity of core muscle endurance. Sisco-Taylor et al. (2022) showed that pain catastrophizing and fear-avoidance behaviors were linked to reduced mobility and functional outcomes. The results of subgroup analysis support these correlations, showing that high PCS and FABQ groups had significantly worse performance-related measures (Saadat et al., 2023). These findings agree with those of Khan (2020) who demonstrated that fear-avoidant attitudes worsen impairmen, and with Varallo et al. (2020), who discussed the inverse relationship between pain catastrophizing and rehabilitation outcomes. Together, these findings highlight the need for a comprehensive understanding of both physical and psychosocial factors to inform the development of therapeutic approaches aimed at improving functional mobility in individuals with CLBP.

### Clinical significance

The findings show that core muscle endurance is the main factor influencing functional performance, emphasizing that core strengthening exercises should be prioritized in rehabilitation programs (Clael et al., 2021). Additionally, psychological barriers such as pain catastrophizing and fear-avoidance attitudes play a significant role in the condition (Ho et al., 2022). This study demonstrates that these factors are connected to mobility, regardless of physical traits, and that they interact with physical characteristics to affect outcomes, supporting integrated patient care. Combining physiological training with cognitive-behavioral therapies may improve functional results and boost quality of life for individuals with CLBP. This information can assist physicians in developing comprehensive treatment plans for this group. Since this was a cross-sectional study, causality cannot be established. Future clinical trials are needed to evaluate the causal effects of combined physical and psychological interventions on both functional and psychological outcomes in people with CLBP.

### Limitations of the study

The cross-sectional study design inherently limits the ability to explore causal relationships between core muscle endurance and functional mobility. Longitudinal studies are necessary to examine these correlations over time and determine the direction of these associations. Additionally, psychosocial factors were evaluated through self-reported measures, which are vulnerable to response bias. Future research could include objective assessments of psychological states or real-time emotional monitoring. Furthermore, although the sample size was adequate for the studies conducted, the results may not be generalizable to populations with different levels of physical activity, comorbidities, or age groups. Including a more diverse demographic and clinical sample would enhance the external validity of the findings. This study did not consider the potential effects of biomechanical or environmental factors, such as gait patterns or occupational demands, on functional mobility. Future research should explore these factors and assess the effectiveness of combined physical and psychosocial interventions on mobility in patients with CLBP. Additional confounders, like pain medication use and environmental influences (*e.g.*, occupational load), were not fully controlled and should be addressed in future longitudinal studies.

## Conclusion

Our findings show that core muscle endurance and psychosocial factors, such as pain catastrophizing and fear-avoidance beliefs, independently predict functional mobility in individuals with CLBP. Core muscle endurance was the strongest physical factor influencing mobility, and psychosocial aspects specifically affected and altered the relationship between core strength and mobility outcomes. These factors are strongly interconnected. Therefore, this underscores the value of an integrated rehabilitation approach that combines core strengthening exercises with treatment of psychological barriers. These results have important practical implications for creating comprehensive treatment plans aimed at improving both mobility and quality of life. Clinically, the findings support multimodal rehabilitation strategies that focus on core strengthening while also addressing psychological distress, especially in patients with high levels of catastrophizing or fear-avoidant behaviors. A key strength of this study is the use of simple, affordable, and validated tools such as core muscle endurance tests and functional mobility assessments, which are easy to implement in routine practice and adaptable to various rehabilitation settings.

## Supplemental Information

10.7717/peerj.20372/supp-1 Supplemental Information 1 Raw Data with Metadata

10.7717/peerj.20372/supp-2 Supplemental Information 2 STROBE checklist

## Additional Information and Declarations

### Data Availability

The following information was supplied regarding data availability: The raw measurements are available in the Supplementary File.
